# Supplementary material for: Comparative intravital imaging of human and rodent malaria sporozoites reveals the skin is not a species‐specific barrier
Source: EMBO Mol Med. 2021 Mar 22;13(4):e11796. doi: 10.15252/emmm.201911796 (PMC8033530; doi:10.15252/emmm.201911796)
Supplement: Supplementary file 1 — Appendix [file EMMM-13-e11796-s011.pdf]

Appendix

Appendix Figure S1. Generation and verification of a *Plasmodium yoelii* line expressing mCherry under control of the *P. berghei csp* promoter

Appendix Table S1. Primers used for genotypic analysis of the Py-mCherry line

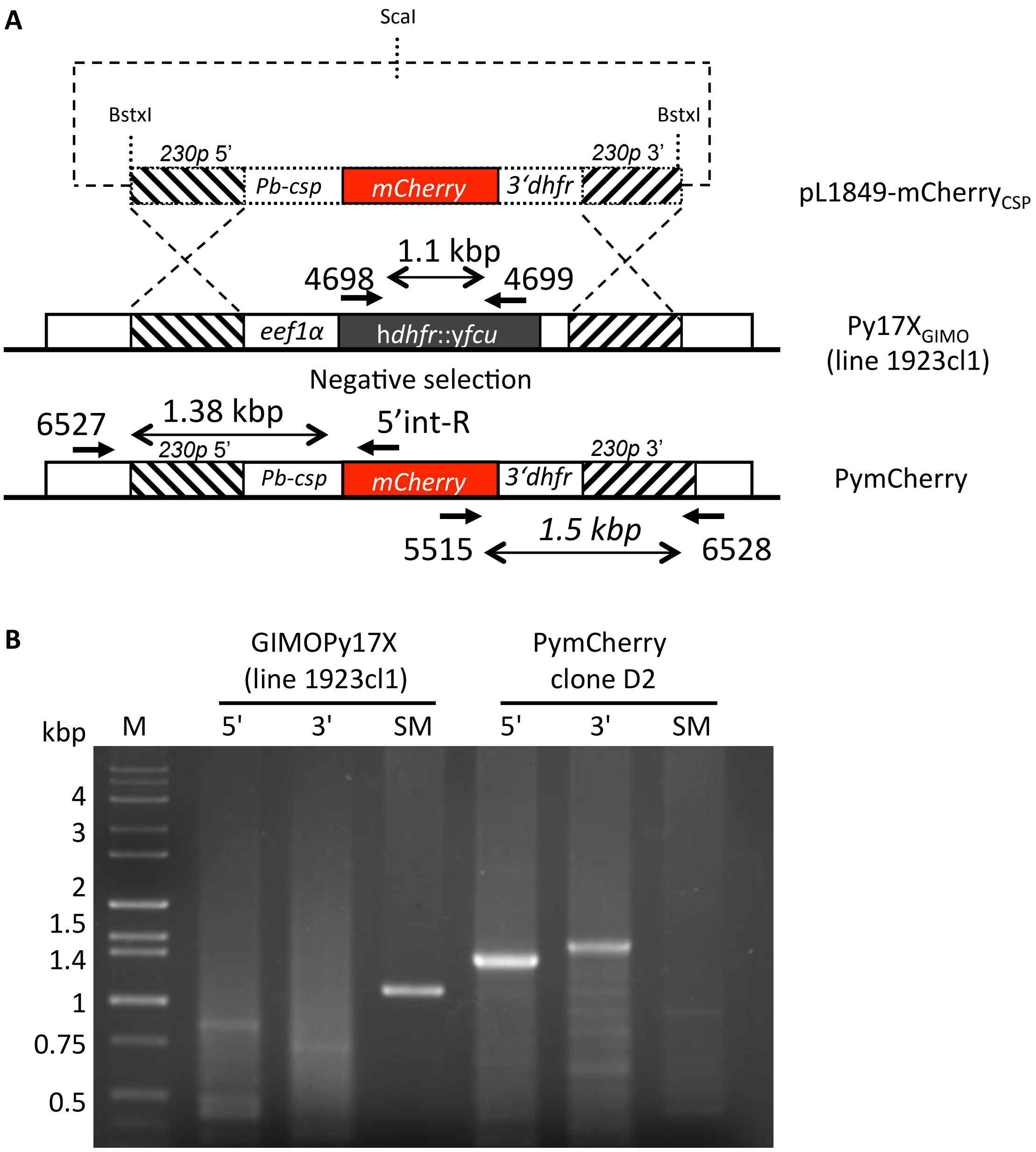

Appendix Figure S1. Generation and verification of a *Plasmodium yoelii* line expressing mCherry under control of the *P. berghei csp* promoter. (A) Schematic representation of the introduction of an mCherry-expression cassette under the control of the *P. berghei csp*-promoter into the Py17X$_{GIMO}$ (line 1923cl1) *(34)*. Transfection construct pL1849-mCherry$_{CSP}$ containing the *pbcsp-mCherry-3'pbdhfr* cassette was linearized by digestion with BstXI and ScaI and was integrated into the modified *P. yoelii* 230p locus containing the h*dhfr*::*yfcu* selectable marker cassette (grey box) by double cross-over homologous recombination at the target regions (hatched boxes). Negative selection with 5-FC selected for parasites that have the mCherry reporter introduced into the genome and the h*dhfr*::*yfcu* marker removed (PymCherry line). Location of primers used for PCR analysis and sizes of PCR products are shown. **(B)** Following transfection and cloning, diagnostic PCRs confirmed integration as expected in the PymCherry line (clone D2), shown by the absence of the h*dhfr*::*yfcu* marker (amplification of h*dhfr*::*yfcu* with primers 4698/4699) and correct 5′- and 3′-integration PCR product sizes (primer pairs 6527/5'int-R and 5515/6528, respectively). See Appendix Table S1 for all primer sequences.

Appendix Table S1. Primers used for genotypic analysis of the Py-mCherry line.

**Generation of Py-mCherry**	
**Primer Name**	**Primer Sequence**
pL0047-PbCS-mCherry-F	GCACGCCCGGGGCCCTTGCGCCCTTAAGACA
pL0047-PbCS-mCherry-R	CGTGCCCCGGGCGAGCTCGGTACCCGAAATTGAA
6527 5'- intgr *py230p*, F	GAAGGATATGAATTAGATCCACC
5'-int-R	ATTGTAAAATTGAGGATGCTTGT
5515 mCherry-F	GCATGGACGAGCTGTACAAG
6528 3'- intgr *py230p*, R	AGACATTGGCATATGAGCAAG
4698 h*dhfr*, F	GTTCGCTAAACTGCATCGTC
4699 y*fcu*, R	GTTTGAGGTAGCAAGTAGACG
