# Supplementary material for: Comparative intravital imaging of human and rodent malaria sporozoites reveals the skin is not a species‐specific barrier
Source: EMBO Mol Med. 2021 Mar 22;13(4):e11796. doi: 10.15252/emmm.201911796 (PMC8033530; doi:10.15252/emmm.201911796)
Supplement: Supplementary file 2 — Expanded View Figures PDF [file EMMM-13-e11796-s010.pdf]

Expanded View Figures

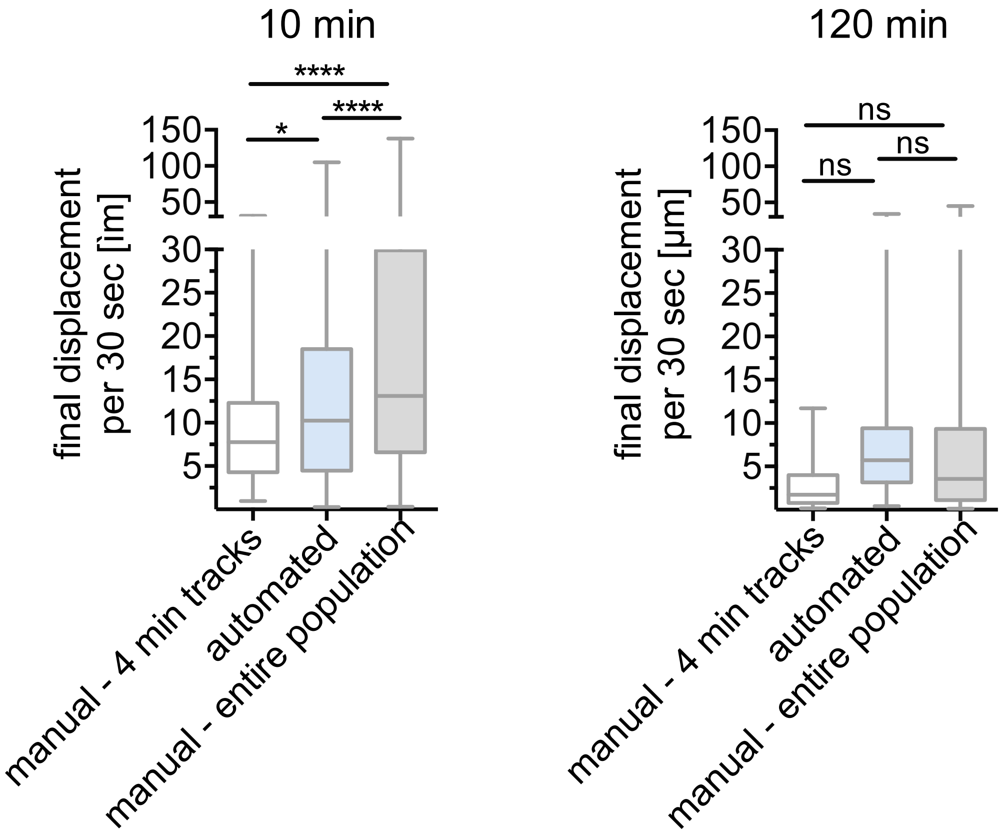

Figure EV1. Sporozoite displacement obtained by tracking of complete 4-min tracks compared to tracking of total sporozoite population.

Displacement obtained by manual tracking of sporozoites that do not leave the field of view throughout the 4-min video, was compared to automated tracking data, corresponding to the entire sporozoite population and data obtained from manual tracking of the entire sporozoite population. Displacement of tracks 10 min (left) and 120 min (right) after intradermal inoculation is shown. Data are displayed in box and whisker plots with horizontal line showing median and the whiskers extending to the minimum and maximum values. A varying number of videos were processed for each time point after inoculation: 10 min (6 videos/74 manual-4 min tracks/292 automated tracks/201 manual-entire population), 120 min (5 videos/48 manual-4 min tracks/151 automated tracks/107 manual-entire population). Statistical analysis, Kruskal–Wallis test, *$P < 0.05$; ****$P < 0.0001$.

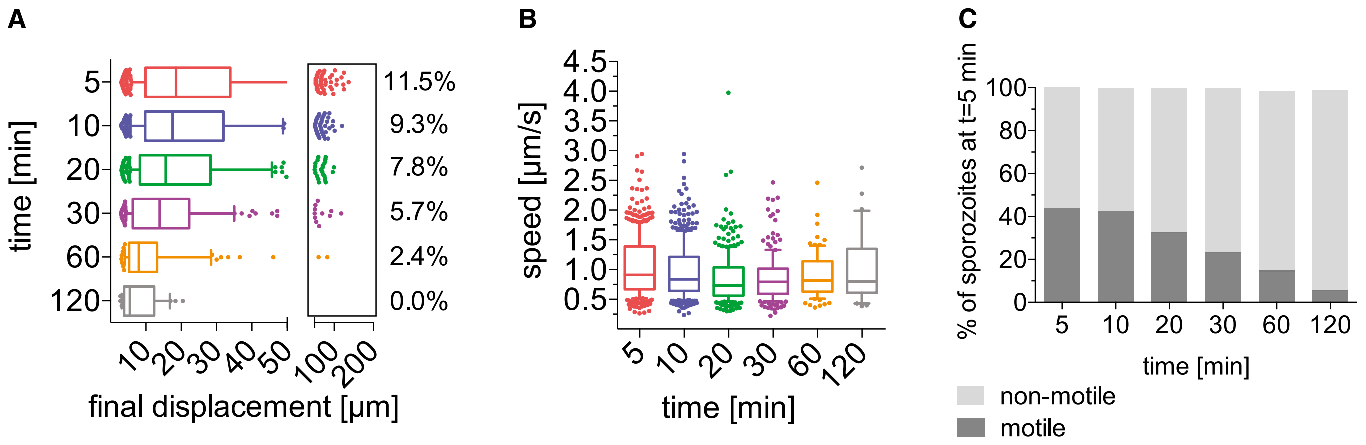

Figure EV2. Motility of *P. falciparum* sporozoites in human skin *ex vivo*.

The data shown originate from four complete imaging sessions from 5 to 120 min after intradermal injection of sporozoites into *ex vivo* human skin tissue.

A Displacement of sporozoites 5–120 min after inoculation. Data are displayed in box and whisker plots with whiskers showing 10–90 percentiles and values below and above the whiskers shown individually. Horizontal lines show the median. Percentage values indicate the fraction of tracks displacing over 75 µm.

B Apparent speed of sporozoites 5 to 120 min after inoculation. Data are displayed in box and whisker plots with whiskers showing 10–90 percentiles and values below and above the whiskers shown individually. Horizontal lines show the median.

C Motile and non-motile sporozoites were manually counted, and proportions are displayed as percentage of sporozoites observed 5 min after inoculation.

Data are pooled from 4 complete time courses.Data information: For panels A and B, 4 videos and a varying number of tracks were processed for each time point: 5 min (4 videos/442 tracks), 10 min (4 videos/428 tracks), 20 min (4 videos/306 tracks), 30 min (4 videos/190 tracks), 60 min (4 videos/82 tracks), and 120 min (4 videos/30 tracks).

 © 2021 The Authors
