# Supplementary material for: Comparative intravital imaging of human and rodent malaria sporozoites reveals the skin is not a species‐specific barrier
Source: EMBO Mol Med. 2021 Mar 22;13(4):e11796. doi: 10.15252/emmm.201911796 (PMC8033530; doi:10.15252/emmm.201911796)
Supplement: Supplementary file 3 — Table EV1 [file EMMM-13-e11796-s012.pdf]

Process	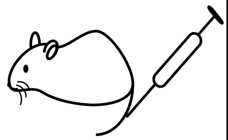	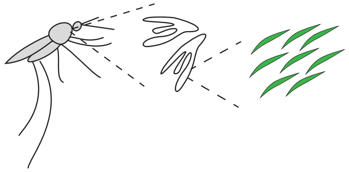	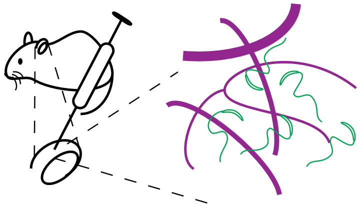	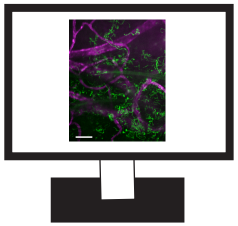
	Antibody injection	Salivary gland dissection<br>Sporozoite isolation	Sporozoite inoculation<br>Intravital imaging	Analysis
Time*	15 minutes	30 minutes	1 hour	Manual analysis of percent motile and blood vessel entry<br>: 2-4 hours<br><br>ICY automated analysis<br>: 30 minutes
Materials	C57Bl6 mice<br>($30/mouse)	Plasmodium infected mosquitoes**	Spinning confocal microscope<br>($29/hour)	Image J and ICY<br>free software

*Time estimates are based on the analysis of one biological replicate for a monoclonal antibody and its relevant control.
**Plasmodium-infected mosquitoes can be obtained from a few laboratories and cost varies.

Table EV1. Steps involved in *in vivo* motility analysis including time and cost estimates.
