# Supplementary material for: Comparative intravital imaging of human and rodent malaria sporozoites reveals the skin is not a species‐specific barrier
Source: EMBO Mol Med. 2021 Mar 22;13(4):e11796. doi: 10.15252/emmm.201911796 (PMC8033530; doi:10.15252/emmm.201911796)
Supplement: Supplementary file 4 — Movie EV1 [file EMMM-13-e11796-s001.zip › Movie_EV1_Legend.docx]

**Movie EV1.** Time-lapse microscopy of *P. berghei* sporozoites (green) starting 10 min after intradermal inoculation, together with CD31-labeled vascular endothelia (magenta). Scale bar, 50 μm. Maximum projection shown in Figure 1A.
