# Supplementary material for: Comparative intravital imaging of human and rodent malaria sporozoites reveals the skin is not a species‐specific barrier
Source: EMBO Mol Med. 2021 Mar 22;13(4):e11796. doi: 10.15252/emmm.201911796 (PMC8033530; doi:10.15252/emmm.201911796)
Supplement: Supplementary file 9 — Movie EV6 [file EMMM-13-e11796-s015.zip › Movie_EV6_Legend.docx]

**Movie EV6.** Time-lapse microscopy of *P. falciparum* sporozoites (green), engaging with CD31-labeled vascular endothelia (magenta). Scale bar, 25 μm. Maximum projection shown in Figure 6C.
