# Supplementary material for: Comparative intravital imaging of human and rodent malaria sporozoites reveals the skin is not a species‐specific barrier
Source: EMBO Mol Med. 2021 Mar 22;13(4):e11796. doi: 10.15252/emmm.201911796 (PMC8033530; doi:10.15252/emmm.201911796)
Supplement: Supplementary file 10 — Movie EV7 [file EMMM-13-e11796-s003.zip › Movie_EV7_Legend.docx]

**Movie EV7.** Time-lapse microscopy showing entry into CD31-labeled blood vessels (magenta) by *P. yoelii* sporozoites (green). Scale bar, 50 μm.
