# Supplementary material for: Comparative intravital imaging of human and rodent malaria sporozoites reveals the skin is not a species‐specific barrier
Source: EMBO Mol Med. 2021 Mar 22;13(4):e11796. doi: 10.15252/emmm.201911796 (PMC8033530; doi:10.15252/emmm.201911796)
Supplement: Supplementary file 14 — Movie EV11 [file EMMM-13-e11796-s014.zip › Movie_EV11_Legend.docx]

**Movie EV11**. Time-lapse microscopy showing *P. falciparum* sporozoites (green) entering both human CD31-labeled vessels (magenta) and unlabeled mouse vessels in a human skin graft. Scale bar, 50 μm.
